# Recommendations for a national Coronavirus disease 2019 response guideline for the care of older persons in Nigeria during and post-pandemic: A family physician’s perspective

**DOI:** 10.4102/phcfm.v12i1.2512

**Published:** 2020-08-11

**Authors:** Olawunmi Olagundoye, Oluwayemisi Enema, Adunola Adebowale

**Affiliations:** 1 Department of Family Medicine, General Hospital Lagos, Lagos Island, Nigeria; 2 General Outpatient Department, Orile Agege General Hospital, Agege, Nigeria; 3 General Outpatient Department, Akodo General Hospital, Ibeju Lekki, Nigeria

**Keywords:** COVID-19, the elderly, prevention, national health policy, older persons

## Abstract

The older persons in our society are a special group of people in need of additional measures of care and protection. They have medical, financial, emotional and social needs. The novel Coronavirus disease 2019 (COVID-19) only exacerbates those needs. COVID-19 is a new disease, and there is limited information regarding the disease. Based on currently available information, older persons and people of any age who have serious underlying medical conditions may be at higher risk of severe illness from COVID-19. Family physicians provide care for individuals across their lifespan. Because geriatricians are internists or family physicians with post-residency training in geriatric medicine, they are major stakeholders in geriatric care. The authors are concerned about the absence of a COVID-19 response guideline/special advisory targeting the vulnerable population of older adults. The management and response to COVID-19 will be implemented in part based on the local context of available resources. Nigeria has been described as a resource-constrained nation. Infection prevention in older persons in Nigeria will far outweigh the possibilities of treatment given limited resources. The aim was to recommend actionable strategies to prevent COVID-19-related morbidity or mortality among older persons in Nigeria and to promote their overall well-being during and after the pandemic. These recommendations cut across the geriatric medicine domains of physical health, mental health, functioning ability and socio-environmental situation.

## Background

The older persons have been defined by the United Nations as those who are 60 years and older.^1^ In a developing country like Nigeria, the same definition applies.^2,3^ The population of older persons in Nigeria is reported to be increasing rapidly.^4^

Their health profile is characterised by decreased immunity, multimorbidity, chronic illnesses, declining physical functions and impaired socialisation necessitating increased demand on healthcare services.^2,4^

Unfortunately, a serious combination of the risk factors that have been identified to portend a worse prognosis or manifestations of severe forms of the Coronavirus disease 2019 (COVID-19) infection is found in older persons. These factors include older age; underlying health problems, often as multimorbidities; and decreased immune status.^5,6^

Cumulative data from the Nigeria Centre for Disease Control (NCDC) on COVID-19 report the highest burden of disease in the age groups spanning 31–60 years. However, the burden of deaths because of COVID-19 is highest amongst elderly persons (60 years and above).^7^ This confirms their vulnerability.

## Justification for a Coronavirus disease 2019 response guideline for the care of older persons

Whilst fever and respiratory symptoms have been widely recognised as key symptoms associated with COVID-19, these symptoms often present differently in older adults. For instance, fever may be blunted or absent in infected older persons. Respiratory symptoms may either be masked or exacerbated by co-occurring diseases, such as chronic obstructive pulmonary disease or congestive cardiac failure, which can further worsen outcomes.^8^ Additional consideration of the peculiarities of the older persons during the COVID-19 pandemic will be useful for healthcare workers in the management of this age group.

The COVID-19 pandemic requires measures such as physical distancing, avoidance of social and religious gatherings and self-isolation if indicated. These measures may adversely impact the emotional, social and financial support of older persons. These negative consequences should be factored in to achieve adequate care of older persons during and after the pandemic.

Given their high risk for the severe manifestation of the disease and a higher rate of mortality, a special advisory/guideline dedicated to this population group during COVID-19 should be made available on the NCDC website similar to the ones that already exist, such as advisory for pregnant women, guidelines for children and nursing mothers, advisory for Ramadan and guidelines for isolation.

## Recommended strategies for the prevention of Coronavirus disease 2019-related morbidity/mortality and the promotion of overall well-being of older persons

1. Keeping older persons who require ongoing chronic care for chronic illnesses away from health facilities as much as possible. This may be achieved in part through the deployment of telemedicine or phone-a-doctor on dedicated toll-free phone lines by establishing remote access to healthcare for non-emergency cases. In this circumstance, care is coordinated as the doctor regulates the physical contact of the elderly with the health facility based on sound clinical judgement. In settings where telemedicine may not be feasible, adults’ clinics can operate a triage system that prioritises older persons for prompt consultation on arrival to reduce their waiting time at healthcare facilities.
2. Adoption of a testing policy that prioritises the screening of persons above 60 years for Severe acute respiratory syndrome coronavirus 2 (SARS-CoV-2) because of the possibility of atypical presentations that are not accounted for in the current definition of suspected cases. This requires a heightened index of suspicion and prompt testing of older persons requiring hospital admissions for other morbidities that may mask the presence of the novel Coronavirus infection or be worsened by co-morbid COVID-19. In essence, older persons requiring hospital admission for other ailments should be tested for COVID-19 and their samples should be prioritised to ensure the shortest turnaround time between sample collection and results.
3. Prioritising the elderly in the distribution of welfare packages/palliatives. Government’s social intervention programmes such as the distribution of ₦20 000 ($51.55) to the poorest of the poor based on pensioners data and food items distribution on the streets may leave some older persons disadvantaged. Pensioners’ associations may be involved in ensuring that the packages get to their members. Additionally, non-governmental organisations should channel a portion of their relief packages directly to senior citizens in various communities by engaging with the community and religious leaders.
4. Taking cognisance of those problems that older persons are predisposed to (dependence, isolation, and depression) and how some of the current COVID-19-related safety precautions (physical distancing and self-isolation of exposed persons) may adversely accentuate these problems. This should be matched with the provision of professional psychological support through appropriate means in the light of the COVID-19 pandemic.
5. Sensitising families and communities to their responsibilities towards older persons during this period. The emphasis should include the following: (1) Provision of food items, household essentials and refill of medications for chronic health conditions; (2) Provision of social and emotional support by maintaining regular communication via phone calls; (3) Communicating the facts about COVID-19 to the less literate older persons without creating panic; (4) Providing the necessary items for Infection Prevention and Control (IPC); (5) Avoiding physical contact in a social situation by ensuring physical distancing (at least 1 m/3 feet) between older persons and their grandchildren, this should be preceded by the proper education of both older children and older persons, and physical distancing may also preclude visits between older persons and their grandchildren; (6) Addressing the subject of surrogate decision-making or right of attorney in the event of unforeseen circumstances amongst other things.
6. Inclusion of the older persons/senior citizens amongst the selected population groups that deserve specific guidelines on the NCDC website addressing their peculiar needs during the COVID-19 pandemic.
7. Post COVID-19 pandemic attention should be given to broadening the scope of health insurance with the inclusion of home-based care in the health insurance schemes at the national and state levels. This will be an improvement on the current status of the national health insurance scheme that emphasises health facility care as the present policy is silent about home-based care.^9^

## Conclusion

We must revise our practices around the care of older persons during this pandemic, both clinically and socially, to ensure that our most vulnerable populations are protected.^8^
